# Changes in estimated glucose disposal rate and future stroke risk in individuals with cardiovascular-kidney-metabolic syndrome stages 0–3

**DOI:** 10.1038/s41598-026-46225-2

**Published:** 2026-04-09

**Authors:** Xinran Wang, Sirun Qin, Bohao Peng, Hong Xiang, Ye Tao, Chengxian Guo, Hongwei Lu

**Affiliations:** 1 Department of Cardiology, The Third Xiangya Hospital, Central South University, Changsha, China; 2 Center for Experimental Medicine, The Third Xiangya Hospital, Central South University, Changsha, China; 3 Department of Nephrology, The Third Xiangya Hospital, Central South University, Changsha, China; 4 Center of Clinical Pharmacology, The Third Xiangya Hospital, Central South University, Changsha, China; 5 Department of Cardiology, Renji Hospital, School of Medicine, Shanghai Jiao Tong University, Shanghai, China; 6 Department of Breast and Thyroid Surgery, The Second Affiliated Hospital of Chongqing Medical University, Chongqing, China

**Keywords:** Stroke, Cardiovascular-kidney-metabolic syndrome, Estimated glucose disposal rate, Insulin resistance, Diseases, Neurology, Neuroscience, Risk factors

## Abstract

Previous studies have reported an association between estimated glucose disposal rate (eGDR) and stroke in individuals with cardiovascular-kidney-metabolic (CKM) syndrome stages 0–3. However, the association of changes in eGDR with the risk of incident stroke in this population remains unclear. Using data from the China Health and Retirement Longitudinal Study (CHARLS), this study included 3849 participants with CKM syndrome stages 0–3, among whom 285 (7.4%) developed stroke during follow-up from 2015 to 2020. Logistic regression was employed to evaluate the impact of cumulative eGDR (cumeGDR) and clusters of eGDR changes on stroke risk. After adjusting for potential confounders, the risk of incident stroke was significantly higher in participants with persistently moderately low eGDR (Class 2: OR 1.51, 95% CI 1.02–2.26), persistently low eGDR (Class 3: OR 2.11, 95% CI 1.36–3.26), and markedly declining eGDR (Class 4: OR 1.78, 95% CI 1.20–2.66), compared with those with persistently high eGDR (Class 1). Lower cumeGDR levels were independently associated with a higher risk of stroke events. Restricted cubic spline analysis indicated a negative linear relationship between cumeGDR and stroke risk. Receiver operating characteristic (ROC) curve analysis demonstrated that cumeGDR had greater predictive value for stroke than eGDR, and incremental predictive value analysis revealed that incorporating cumeGDR or clusters of eGDR changes into the baseline model provided incremental value for stroke risk prediction. These findings suggest that among individuals with CKM syndrome stages 0–3, persistently low eGDR or markedly declining eGDR was associated with a higher risk of stroke, underscoring the clinical value of dynamic eGDR monitoring for the early identification of individuals at high risk for stroke.

**Supplementary Information:**

The online version contains supplementary material available at 10.1038/s41598-026-46225-2.

## Introduction

Stroke stands as a major contributor to global mortality, which not only leads to significant health losses but also drives a notable increase in both disease burden and economic costs for healthcare systems^1,2^. Despite remarkable progress made in the prevention, diagnosis, and management of stroke in recent years, the disease burden associated with this condition still proves hard to rein in^3,4^. The American Heart Association (AHA) introduced the concept of the cardiovascular-kidney-metabolic (CKM) syndrome in October 2023, defining it as a systemic condition characterized by the interplay among metabolic risk factors, chronic kidney disease (CKD), and cardiovascular disease (CVD)^5^. This complex interplay markedly increases the susceptibility to adverse CVD outcomes, encompassing stroke^5,6^. This highlights the need to strengthen preventive strategies in individuals with CKM syndrome, with the aim of halting progression to stroke.

Insulin resistance (IR) reduces tissue responsiveness to insulin, leading to abnormalities in glucose and lipid metabolism, and plays a key role in the development and progression of CKM syndrome^5,7^. IR has been shown to be closely associated with the development of CVD^7–9^. It increases CVD risk by contributing to metabolic disorders, endothelial dysfunction, and inflammatory states^7,8,10^. Therefore, quantifying and monitoring IR is of particular importance. The gold standard test for measuring IR is the euglycemic-hyperinsulinemic clamp^11^. Nevertheless, the method is invasive, time-consuming, and expensive, restricting its use in large-scale clinical settings. Calculated from waist circumference (WC), hypertension, and glycated hemoglobin (HbA1c), the estimated glucose disposal rate (eGDR) is a derived surrogate marker of IR^12,13^. It has been widely used in epidemiological research as a practical alternative to direct assessments of insulin sensitivity^14^. Lower eGDR values indicate a higher degree of IR^15^. Recent studies have identified a close association between the IR index eGDR and stroke risk in individuals with CKM syndrome stages 0–3^16,17^. However, these studies lack systematic analyses into the association of changes in eGDR with stroke risk. Relying solely on eGDR measured at a single time point fails to capture the dynamic evolution of IR. Multiple time-point measurements of eGDR more accurately represent the dynamic progression of IR.

Hence, we analyzed data obtained from the China Health and Retirement Longitudinal Study (CHARLS) to elucidate the association of cumulative eGDR (cumeGDR) and clusters of eGDR changes with stroke risk in individuals with CKM syndrome stages 0–3, with the aim of providing new insights for early intervention and prevention strategies for stroke in this population.

## Methods

### Study population

Data were obtained from CHARLS, a large, nationally representative cohort of Chinese adults aged ≥ 45 years. Using a multistage, stratified probability sampling design, 17,708 participants were enrolled in the baseline survey (Wave 1) conducted between 2011 and 2012 across 28 provinces, 150 counties, and 450 communities. Four follow-up waves were completed by 2020 (Wave 2 in 2013, Wave 3 in 2015, Wave 4 in 2018, and Wave 5 in 2020). The previous report has provided detailed descriptions of the CHARLS study design and data collection procedures^18^. The CHARLS protocol complied with the Declaration of Helsinki and received approval from the Biomedical Ethics Review Committee of Peking University (IRB 00001052–11015). All participants gave written informed consent.

The selection process of the study population is shown in Figure S1. Among 17,708 participants, 13,859 were excluded due to the following criteria: (1) age under 45 years, (2) stroke was present before Wave 3 or stroke information was incomplete or lost to follow-up, (3) eGDR was unavailable at Wave 1 or Wave 3, (4) CKM stages could not be determined or were stage 4, and (5) non-fasting. Ultimately, 3849 eligible participants were included in the analysis.

### Assessment of eGDR

The eGDR (mg/kg/min) was computed according to the following equation: eGDR = 21.158 − (0.09 × WC) − (3.407 × HT) − (0.551 × HbA1c), where WC represents waist circumference (cm), HT indicates hypertension (yes = 1, no = 0), and HbA1c is glycated hemoglobin (%). The cumeGDR was estimated according to the following equation: cumeGDR = (eGDR_2012_ + eGDR_2015_)/2 × time_(2015–2012)_^19^.

### Assessment of CKM syndrome stages 0–4

The staging of CKM syndrome is defined as follows: Stage 0, no CKM risk factors; Stage 1, excessive or dysfunctional obesity; Stage 2, metabolic disorders such as hypertriglyceridemia, hypertension, diabetes, or metabolic syndrome, or moderate-to-high-risk CKD; Stage 3, subclinical CVD in the context of CKM syndrome or its risk equivalents; and Stage 4, clinical CVD in the context of CKM syndrome, including coronary heart disease, heart failure, atrial fibrillation, stroke, and other related conditions^5^.

### Assessment of stroke

The outcome of this study was the incidence of stroke during follow-up. Stroke events were identified based on self-reported physician diagnoses. The CHARLS research team implemented strict quality control procedures for data recording and verification to ensure reliability^18^.

### Data collection

Demographic characteristics, physical examinations, health status, and laboratory measurements were collected from all participants. Demographic characteristics included age, sex, residence, educational level, marital status, alcohol consumption, and smoking status. Physical examinations and health status included systolic blood pressure (SBP), diastolic blood pressure (DBP), waist circumference (WC), body mass index (BMI), and histories of hypertension, diabetes, and dyslipidemia. Laboratory measurements included high-density lipoprotein cholesterol (HDL-C), low-density lipoprotein cholesterol (LDL-C), triglycerides (TG), total cholesterol (TC), glycated hemoglobin (HbA1c), C-reactive protein (CRP), uric acid (UA), serum creatinine (Scr), and fasting blood glucose (FBG). Participants were considered hypertensive if they had a history of hypertension, were taking antihypertensive medication, or had an SBP ≥ 130 mmHg or DBP ≥ 80 mmHg^16,20^. Participants were considered diabetic if they had a history of diabetes, were taking antidiabetic medication, or had an FBG ≥ 126 mg/dL or HbA1c ≥ 6.5%^16,21^.

### Statistical analysis

Continuous variables with a normal distribution were presented as mean ± standard deviation, while non-normally distributed continuous variables were expressed as median (interquartile range). Categorical variables were reported as counts (percentages). One-way ANOVA was used for normally distributed continuous variables, and the Kruskal–Wallis test was applied for non-parametric data. The χ² test was used for categorical variables. Missing values were imputed using multiple imputation (Table S1).

**Fig. 1:**
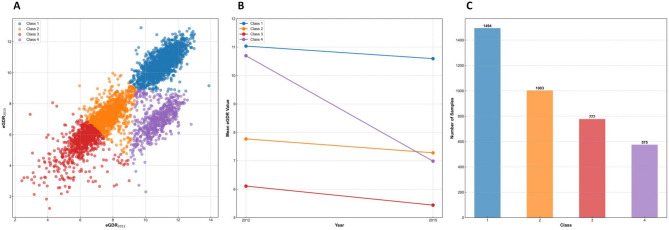
Cluster analysis of eGDR changes from 2012 to 2015. (**A**) Clustering diagrams of eGDR changes based on K-means; (**B**) eGDR changes in each cluster; (**C**) number of participants in each cluster.

Based on eGDR measurements in 2012 and 2015, participants were grouped into trajectory patterns using K-means clustering. Before clustering, eGDR values were standardized using z-score transformation to eliminate potential scale-related bias. The optimal number of clusters (K = 4) was determined using both the elbow method and the Silhouette coefficient to ensure the stability and rationality of the clustering results (Figure S2). Participants were finally categorized into four eGDR control levels (Fig. 1): Class 1 (*n* = 1494, 38.82%), eGDR decreased slightly from 11.03 ± 0.76 in 2012 to 10.59 ± 0.79 in 2015, with an absolute change of − 0.44, representing persistently high eGDR; Class 2 (*n* = 1003, 26.06%), eGDR decreased from 7.77 ± 0.68 to 7.28 ± 0.78, with an absolute change of − 0.49, representing persistently moderately low eGDR; Class 3 (*n* = 777, 20.19%), eGDR decreased from 6.11 ± 0.89 to 5.44 ± 0.95, with an absolute change of − 0.67, representing persistently low eGDR; Class 4 (*n* = 575, 14.94%), eGDR declined from 10.70 ± 0.79 to 6.99 ± 0.98, with an absolute change of − 3.71, representing markedly declining eGDR.

Participants were also divided into three groups according to tertiles of cumeGDR: low (T1), medium (T2), and high (T3) groups.

Logistic regression analysis was used to evaluate the association between cumeGDR or clusters of eGDR changes and stroke incidence, and odds ratios (ORs) with 95% confidence intervals (CIs) were calculated for four models. Model 1 was unadjusted. Model 2 was adjusted for age and sex. Model 3 was further adjusted for age, sex, residence, educational level, marital status, alcohol consumption, and smoking status. Model 4 included all variables from Model 3 and was further adjusted for SBP, DBP, BMI, eGFR, TG, HDL-C, LDL-C, CRP, UA, and FBG. Multicollinearity among variables was assessed, and all variance inflation factors (VIFs) were below 5, indicating no significant multicollinearity (Table S2). Restricted cubic spline (RCS) regression models were used to explore potential non-linear associations between cumeGDR and stroke events in the total CKM stages 0–3 population and in different CKM stages. Receiver operating characteristic (ROC) curve analysis was conducted to evaluate the diagnostic value, and the area under the curve (AUC) was calculated to quantify the predictive ability of eGDR and cumeGDR for stroke. Incremental predictive value analyses were used to determine whether cumeGDR and the cluster of eGDR changes could improve risk reclassification and discrimination for stroke. Subgroup and interaction analyses were conducted to investigate the association between cumeGDR or clusters of eGDR changes and stroke occurrence across different demographic characteristics, including age, sex, residence, education, smoking, drinking status, and CKM stages. To rigorously assess the robustness of the findings, sensitivity analyses were performed by excluding participants with imputed data and those who developed stroke within 3 years.

All statistical analyses were performed using R software (version 4.5.1; https://www.r-project.org/) or Python (version 3.13.7; https://www.python.org/). Two-sided *P* values < 0.05 were considered statistically significant.

## Results

### Baseline characteristics of participants

A total of 3849 participants were included in this study. The mean age at baseline was 58.43 ± 8.69 years, with 1,817 males (47.21%). The numbers of participants at CKM stages 0, 1, 2, and 3 were 255 (6.63%), 724 (18.81%), 1,792 (46.56%), and 1,078 (28.01%), respectively. The mean eGDR was 9.14 ± 2.17 in 2012 and 8.15 ± 2.22 in 2015, with cumeGDR of 25.94 ± 6.24. Baseline characteristics by eGDR cluster are shown in Table 1. Overall, compared with Class 1, participants in the other classes were older, had lower rates of marriage and education, and exhibited higher SBP, DBP, WC, BMI, CRP, LDL-C, TC, TG, UA, FBG, and HbA1c, along with lower HDL-C and eGFR levels. Baseline characteristics of participants grouped by tertiles of cumeGDR are presented in Supplementary Table S3.

**Table 1:** Characteristics of the study participants based on clusters of eGDR changes.

Characteristic	Overall (*n* = 3849)	The eGDR cluster groups				*P* value
		Class 1	Class 2	Class 3	Class 4	
		(*n* = 1494)	(*n* = 1003)	(*n* = 777)	(*n* = 575)	
Age, years	58.43 ± 8.69	56.51 ± 8.08	60.49 ± 9.06	59.82 ± 8.69	57.93 ± 8.40	< 0.001
Gender, n (%)						0.046
Male	1817 (47.21)	699 (46.8)	486 (48.5)	339 (43.6)	293 (51)	
Female	2032 (52.79)	795 (53.2)	517 (51.5)	438 (56.4)	282 (49)	
Marital status, n (%)						< 0.001
Married	3478 (90.36)	1381 (92.4)	874 (87.1)	694 (89.3)	529 (92)	
Others	371 (9.64)	113 (7.6)	129 (12.9)	83 (10.7)	46 (8)	
Education, n (%)						< 0.001
Primary school or below	2696 (70.04)	995 (66.6)	753 (75.1)	545 (70.1)	403 (70.1)	
Above primary school	1153 (29.96)	499 (33.4)	250 (24.9)	232 (29.9)	172 (29.9)	
Habitation, n (%)						< 0.001
Agriculture	2598 (67.5)	1037 (69.4)	706 (70.4)	466 (60)	389 (67.7)	
Others	1251 (32.5)	457 (30.6)	297 (29.6)	311 (40)	186 (32.3)	
Smoking status, n (%)						0.001
Never	2360 (61.31)	926 (62)	580 (57.8)	508 (65.4)	346 (60.2)	
Previous	289 (7.51)	95 (6.4)	79 (7.9)	72 (9.3)	43 (7.5)	
Current	1200 (31.18)	473 (31.7)	344 (34.3)	197 (25.4)	186 (32.3)	
Drinking status, n (%)						< 0.001
Never	2251 (58.48)	908 (60.8)	554 (55.2)	464 (59.7)	325 (56.5)	
Previous	284 (7.38)	91 (6.1)	88 (8.8)	73 (9.4)	32 (5.6)	
Current	1314 (34.14)	495 (33.1)	361 (36)	240 (30.9)	218 (37.9)	
Comorbidities, n (%)						
Diabetes	552 (14.34)	104 (7)	150 (15)	232 (29.9)	66 (11.5)	< 0.001
Dyslipidemia	297 (7.72)	75 (5)	70 (7)	126 (16.2)	26 (4.5)	< 0.001
SBP, mmHg	130.87 ± 21.37	115.98 ± 11.00	144.14 ± 20.04	148.15 ± 20.75	123.04 ± 10.65	< 0.001
DBP, mmHg	76.3 ± 12.46	69.13 ± 8.72	82.12 ± 12.24	85.12 ± 12.06	72.86 ± 8.65	< 0.001
WC, cm	84.94 ± 9.7	81.14 ± 7.95	82.78 ± 8.46	95.52 ± 7.07	84.30 ± 8.69	< 0.001
BMI, kg/m^2^	23.32 ± 3.48	22.11 ± 2.88	22.53 ± 3.11	26.78 ± 3.02	23.2 ± 3.09	< 0.001
CRP, mg/L	0.96 [1.42]	0.81[1.20]	0.95[1.39]	1.41[1.93]	0.91[1.27]	< 0.001
HDL, mg/dL	51.43 ± 15.17	53.18 ± 14.93	52.99 ± 15.80	45.40 ± 12.47	52.32 ± 15.93	< 0.001
LDL, mg/dL	117.16 ± 34.77	114.71 ± 32.18	116.72 ± 34.69	122.95 ± 38.28	116.49 ± 35.60	< 0.001
TC, mg/dL	194.04 ± 37.93	188.64 ± 36.24	194.98 ± 36.26	203.42 ± 40.40	193.77 ± 39.14	< 0.001
TG, mg/dL	101.78 [74.34]	92.04[60.18]	100.89[74.34]	135.4[96.46]	98.24[74.78]	< 0.001
BUN, mg/dL	15.66 ± 4.35	15.68 ± 4.31	15.84 ± 4.47	15.61 ± 4.24	15.39 ± 4.41	0.253
UA, mg/dL	4.36 ± 1.21	4.19 ± 1.12	4.40 ± 1.21	4.65 ± 1.31	4.35 ± 1.20	< 0.001
FBG, mg/dL	108.37 ± 32.95	101.15 ± 18.05	108.84 ± 29.46	124.14 ± 54.07	105.03 ± 21.92	< 0.001
HbA1c, %	5.27 ± 0.8	5.11 ± 0.43	5.23 ± 0.82	5.70 ± 1.25	5.14 ± 0.48	< 0.001
eGFR, mL/min/1.73 m^2^	92.22 ± 15.47	94.31 ± 14.19	90.11 ± 16.28	89.55 ± 16.60	94.07 ± 14.62	< 0.001
CKM stage, n (%)						< 0.001
0	255 (6.63)	211 (14.1)	2 (0.2)	0 (0)	42 (7.3)	
1	724 (18.81)	578 (38.7)	19 (1.9)	7 (0.9)	120 (20.9)	
2	1792 (46.56)	505 (33.8)	581 (57.9)	435 (56)	271 (47.1)	
3	1078 (28.01)	200 (13.4)	401 (40)	335 (43.1)	142 (24.7)	
eGDR2012	9.14 ± 2.17	11.03 ± 0.76	7.77 ± 0.68	6.11 ± 0.89	10.70 ± 0.79	< 0.001
eGDR2015	8.15 ± 2.22	10.59 ± 0.79	7.28 ± 0.78	5.44 ± 0.95	6.99 ± 0.98	< 0.001
CumeGDR	25.94 ± 6.24	32.44 ± 2.14	22.58 ± 1.84	17.32 ± 2.32	26.53 ± 2.26	< 0.001
eGDR absolute change	−0.99	−0.44	−0.49	−0.67	−3.71	< 0.001
Stroke, n (%)	285 (7.4)	64 (4.3)	80 (8)	95 (12.2)	46 (8)	< 0.001

SBP, systolic blood pressure; DBP, diastolic blood pressure; WC, waist circumference; BMI, body mass index; CRP, C-reactive protein; HDL-C, high-density lipoprotein cholesterol; LDL-C, low-density lipoprotein cholesterol; TC, total cholesterol; TG, triglyceride; BUN, blood urea nitrogen; UA, uric acid; FBG, fasting blood glucose; HbA1c, glycated hemoglobin; eGFR, estimated glomerular filtration rate; eGDR, estimated glucose disposal rate; CumeGDR, cumulative estimated glucose disposal rate;.

### The association of cumeGDR and clusters of eGDR changes with stroke risk in individuals with CKM syndrome stages 0–3

**Fig. 2:**
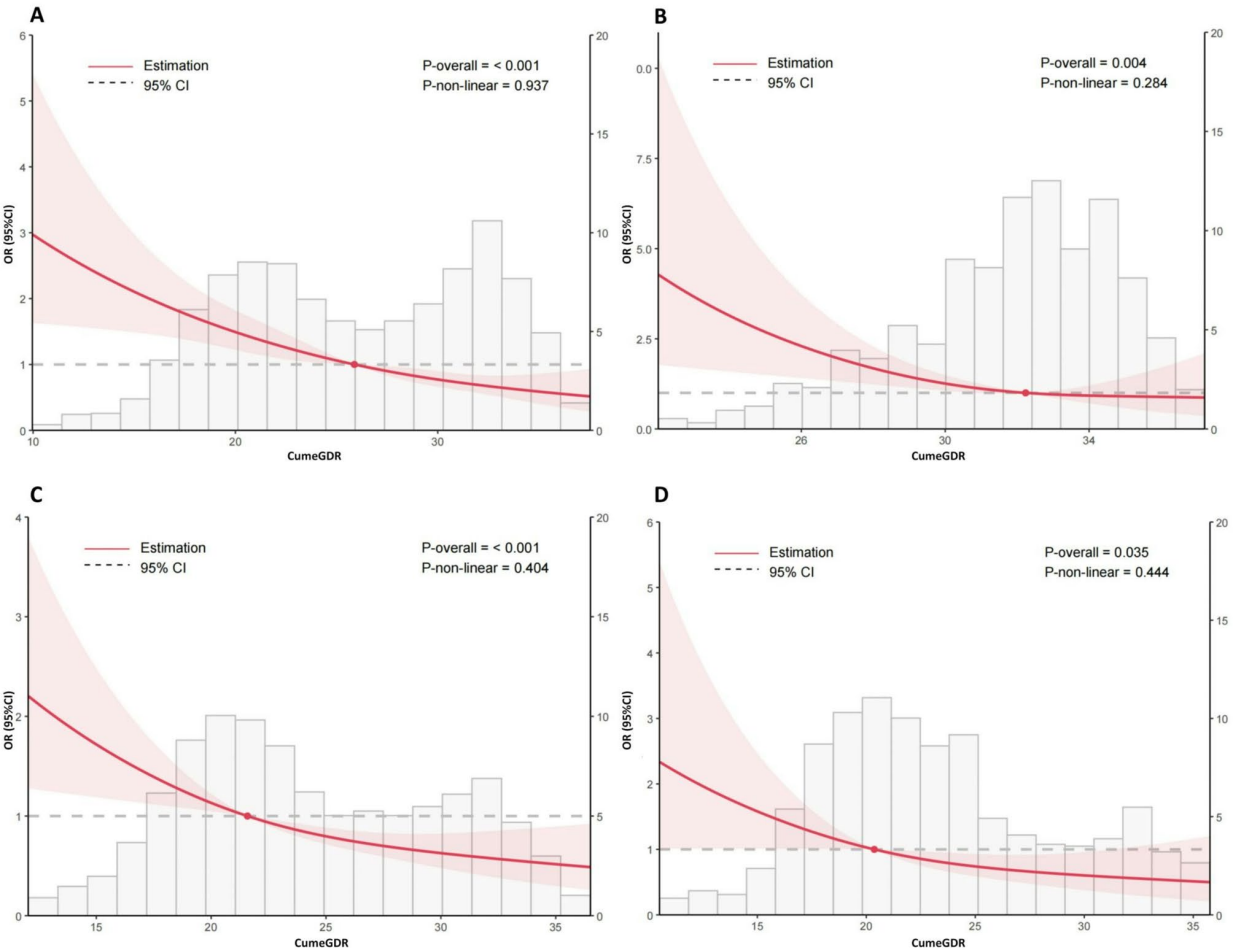
Restricted cubic splines analysis between cumeGDR and stroke incidence in a population with CKM syndrome stages 0–3 (**A**), stages 0–1 (**B**), stages 2 (**C**) and stages 3 (**D**). The analysis was adjusted using Model 4.

During the follow-up period from 2015 to 2020, 285 participants (7.4%) developed stroke. Logistic regression models were used to assess the association of cumeGDR and clusters of eGDR changes with stroke risk in individuals with CKM syndrome stages 0–3. The results showed that cumeGDR and clusters of eGDR changes were significantly associated with stroke risk, and this association remained robust following full adjustment (Table 2). In the fully adjusted Model IV, compared with the group with persistently high eGDR levels (Class 1), the risk of incident stroke was significantly higher in the group with persistently moderately low eGDR (Class 2; OR 1.51, 95% CI 1.02–2.26), the group with persistently low eGDR (Class 3; OR 2.11, 95% CI 1.36–3.26), and the group with markedly declining eGDR (Class 4; OR 1.78, 95% CI 1.20–2.66). Furthermore, lower cumeGDR levels were independently associated with higher risk of stroke events. In Model IV, each one-standard-deviation increase in cumeGDR was associated with a 30% reduction in stroke risk. When cumeGDR was divided into tertiles, participants in T2 (OR 2.19, 95% CI 1.49–3.21) and T1 (OR 2.76, 95% CI 1.80–4.25) had significantly higher stroke risk compared with T3. Restricted cubic spline regression showed a negative linear relationship between cumeGDR and stroke risk in individuals with CKM stages 0–3, which was consistent across different CKM stages (Fig. 2).

**Table 2:** Logistic regression analysis for the association between different eGDR groups and stroke.

Variable	Model 1		Model 2		Model 3		Model 4	
	OR (95% CI)	*P* value	OR (95% CI)	*P* value	OR (95% CI)	*P* value	OR (95% CI)	*P* value
Categories								
Class 1	Reference		Reference		Reference		Reference	
Class 2	1.94 (1.38–2.72)	< 0.001	1.78 (1.26–2.51)	< 0.001	1.76 (1.25–2.48)	0.001	1.51 (1.02–2.26)	0.046
Class 3	3.11 (2.24–4.33)	< 0.001	2.91 (2.09–4.06)	< 0.001	2.98 (2.13–4.16)	< 0.001	2.11 (1.36–3.26)	< 0.001
Class 4	1.94 (1.31–2.87)	< 0.001	1.89 (1.27–2.80)	0.002	1.88 (1.27–2.79)	0.002	1.78 (1.20–2.66)	0.004
Tertiles								
T 3	Reference		Reference		Reference		Reference	
T 2	2.44 (1.69–3.52)	< 0.001	2.35 (1.63–3.39)	< 0.001	2.35 (1.63–3.40)	< 0.001	2.19 (1.49–3.21)	< 0.001
T 1	3.59 (2.53–5.10)	< 0.001	3.38 (2.37–4.81)	< 0.001	3.44 (2.41–4.91)	< 0.001	2.76 (1.80–4.25)	< 0.001
*P* for trend		< 0.001		< 0.001		< 0.001		< 0.001
CumeGDR^*^	0.63 (0.56–0.71)	< 0.001	0.64 (0.57–0.73)	< 0.001	0.64 (0.56–0.73)	< 0.001	0.70 (0.59–0.84)	< 0.001

*Per SD.

### Predictive value of cumeGDR and clusters of eGDR changes for stroke

**Fig. 3:**
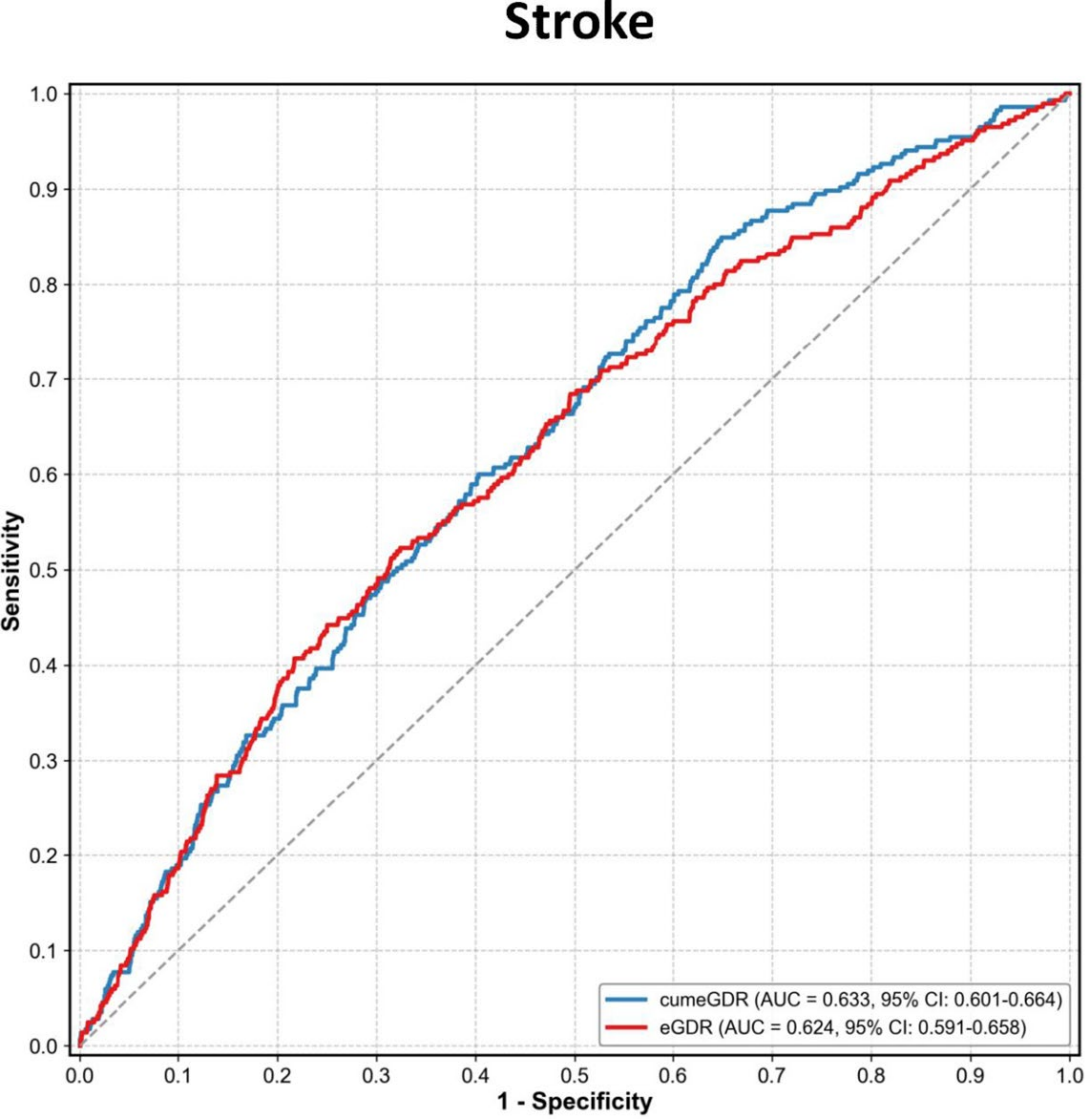
Receiver operating characteristic curves comparing the predictive performance of eGDR and cumeGDR for stroke.

The predictive performance of eGDR and cumeGDR for stroke among individuals with CKM syndrome stages 0–3 was evaluated using ROC curve analysis. Compared to eGDR (AUC 0.624, 95% CI: 0.591–0.658), cumeGDR demonstrated a higher AUC in predicting stroke (AUC 0.633, 95% CI: 0.601–0.664) (Fig. 3). Furthermore, Table S4 presents the incremental predictive value of cumeGDR and clusters of eGDR changes for stroke in individuals with CKM syndrome stages 0–3. After incorporating cumeGDR or clusters of eGDR into the baseline model, the concordance statistic (C-statistic), net reclassification improvement (NRI), and integrated discrimination improvement (IDI) were all improved.

### Subgroup and sensitivity analyses

 Subgroup and interaction analyses were carried out after stratifying the study population by age, sex, residence, education level, smoking and drinking habits, and CKM stages. For the eGDR cluster groups or cumeGDR groups, no significant subgroup interactions were identified (Tables S5–S6). Sensitivity analyses conducted in the complete dataset without multiple imputation or after excluding participants who developed stroke within 3 years yielded consistent results (Tables S7and S8).

## Discussion

This study provides valuable insights into the association of changes in eGDR with the risk of incident stroke in individuals with CKM syndrome stages 0–3. We found that persistently low eGDR or markedly declining eGDR was associated with a higher risk of stroke. Furthermore, cumeGDR demonstrated superior predictive value for stroke compared to a single eGDR measurement, and both cumeGDR and eGDR change clusters provided incremental value for stroke prediction.

The AHA identifies individuals with CKM syndrome stages 0–3 as a priority population for early CVD intervention and highlights the central role of IR in driving CVD risk within this group^5^. IR has been demonstrated to be an independent risk factor for the development of CVD^8,22^. eGDR has shown considerable reliability as a biomarker for evaluating IR^23^. A recent study by Dong et al. focusing on individuals with CKM stages 0–3 demonstrated that lower eGDR levels are associated with a higher risk of heart disease and stroke^16^. Similarly, research by Chen et al. indicated that low eGDR levels are associated with higher mortality in individuals with CKM stages 0–3^24^. These studies collectively highlight the critical value of eGDR in risk assessment among individuals with CKM stages 0–3. However, given that eGDR is a time-varying IR marker, a single measurement cannot fully capture its dynamics. Continuous monitoring of eGDR can more accurately reflect the trajectory of IR in this population.

This study used K-means clustering to systematically investigate the association between clusters of eGDR changes and stroke incidence in individuals with CKM stages 0–3. After adjusting for potential confounders, the results showed that, compared with the persistently high eGDR group (Class 1), the persistently moderately low eGDR group (Class 2), persistently low eGDR group (Class 3), and markedly declining eGDR group (Class 4) all had significantly increased stroke risk. Notably, the markedly declining eGDR group (Class 4) had similar eGDR levels to Class 1 in 2012 and similar levels to Class 2 in 2015, yet it showed a pattern of higher stroke odds relative to Class 2. This suggests that rapid deterioration of IR (rapid eGDR decline) may serve as a critical early warning signal for stroke. Furthermore, the study revealed that cumeGDR is closely associated with stroke risk. After full adjustment, participants in the lowest cumeGDR tertile had the highest stroke risk, and each one standard deviation increase in cumeGDR reduced the risk of stroke by 30%. These findings underscore the importance of dynamically monitoring eGDR in individuals with CKM stages 0–3 and provide key evidence for early identification of high-risk individuals and the development of precise intervention strategies.

Evidence from Dong et al. suggests that eGDR outperformed six commonly used IR indices in predicting CVD events among individuals with CKM syndrome stages 0–3, especially stroke^16^. In addition, He et al. found that eGDR showed favorable predictive performance for all-cause and CVD mortality compared with other widely used IR indices, including the TyG index, TyG-WC, TyG-BMI, TyG-WHtR, and the homeostasis model assessment of IR^25^. The findings of Dong et al. and He et al. collectively underscore the considerable predictive value of eGDR in CVD-related events. From the two critical dimensions of “disease risk” and “mortality risk,” their results consistently demonstrate the prominent role of eGDR in assessing IR-related CVD risk. In this study, we evaluated the predictive value of cumeGDR versus a single eGDR measurement for stroke in individuals with CKM syndrome stages 0–3. CumeGDR was superior to a single eGDR measurement in predicting stroke risk. In addition, we found that both cumeGDR and clusters of eGDR changes provided incremental value for stroke prediction. This suggests that, for individuals with CKM syndrome stages 0–3, dynamic monitoring of eGDR may more reliably reflect future stroke risk than a single measurement.

Stroke is a prevalent and severe CVD complication among individuals with CKM syndrome^5,26^. The development of CVD induced by IR involves several molecular mechanisms. Under IR, insulin cannot fully exert its biological activity, resulting in impaired cellular glucose uptake and utilization, insufficient energy supply, and disrupted tissue metabolism, which collectively promote the onset and progression of CVD^7,27,28^. First, IR directly disrupts glucose metabolic homeostasis, leading to persistent hyperglycemia, which in turn promotes excessive generation of reactive oxygen species (ROS) and abnormal accumulation of advanced glycation end products, thereby exacerbating low-grade inflammation and increasing the risk of CVD^10^. IR can also induce vascular endothelial dysfunction by reducing nitric oxide production in endothelial cells and promoting the release of pro-coagulant factors, ultimately leading to platelet aggregation^7,29^. Meanwhile, IR impairs insulin signaling in adipocytes, which enhances lipolysis and further increases lipid load to the liver^30^. This abnormal hyperlipidemic state, together with altered platelet function, contributes to the formation of atherosclerotic plaques^7,29,30^. Additionally, IR has been found to be associated with vascular smooth muscle cell proliferation and macrophage activation, which are involved in atherosclerotic plaque formation^10,31,32^.

This study has several limitations. First, all participants were middle-aged and elderly adults from China, so the findings may not be directly generalizable to populations from other countries or to other age groups. Future multinational cohort studies could be considered to further explore this field. Second, eGDR was assessed only twice at specific time points, which may not fully capture its dynamic trajectory. Increasing the frequency of measurements in future research could provide a more detailed evaluation of longitudinal changes in eGDR. Third, although multiple confounding factors were adjusted for, residual confounding cannot be entirely excluded. Fourth, although eGDR has been widely used in epidemiological studies as a surrogate marker of IR, it remains a calculated scoring index that may not fully capture the complex pathophysiological mechanisms underlying IR. Finally, CVD was determined based on self-reported physician diagnoses rather than gold-standard clinical assessments, which may introduce misclassification bias. However, previous studies have validated the high reliability of self-reported CVD events, which may reduce the impact of this bias^33,34^.

## Conclusion

Among individuals with CKM syndrome stages 0–3, changes in eGDR were independently associated with the risk of incident stroke. Persistently low eGDR or markedly declining eGDR was linked to a higher risk of stroke. Thus, dynamic monitoring of eGDR may facilitate the early identification of high-risk individuals with CKM syndrome stages 0–3 for incident stroke.

## Supplementary Information

Below is the link to the electronic supplementary material.

Supplementary Material 1

## Data Availability

The datasets generated and/or analyzed during this study are publicly available in the CHARLS repository, [http://charls.pku.edu.cn].

## Abbreviations

eGDR: Estimated glucose disposal rate
CKM: Cardiovascular-kidney-metabolic
AHA: American Heart Association
CKD: Chronic kidney disease
CVD: Cardiovascular disease
IR: Insulin resistance
WC: Waist circumference
HbA1c: Glycated hemoglobin
CHARLS: China Health and Retirement Longitudinal Study
cumeGDR: Cumulative estimated glucose disposal rate
SBP: Systolic blood pressure
DBP: Diastolic blood pressure
BMI: Body mass index
HDL-C: High-density lipoprotein cholesterol
LDL-C: Low-density lipoprotein cholesterol
TG: Triglycerides
TC: Total cholesterol
CRP: C-reactive protein
UA: Uric acid
Scr: Serum creatinine
FBG: Fasting blood glucose
OR: Odds ratio
CI: Confidence interval
ROC: Receiver operating characteristic
AUC: Area under the curve
RCS: Restricted cubic spline
VIF: Variance inflation factor
eGFR: Estimated glomerular filtration rate
ANOVA: Analysis of variance
C-statistic: Concordance statistic
NRI: Net reclassification improvement
IDI: Integrated discrimination improvement
ROS: Reactive oxygen species
