# Supplementary material for: Changes in estimated glucose disposal rate and future stroke risk in individuals with cardiovascular-kidney-metabolic syndrome stages 0–3
Source: Sci Rep. 2026 Apr 9;16:16737. doi: 10.1038/s41598-026-46225-2 (PMC13223218; doi:10.1038/s41598-026-46225-2)
Supplement: Supplementary file 1 — Supplementary Material 1 [file 41598_2026_46225_MOESM1_ESM.docx]

**
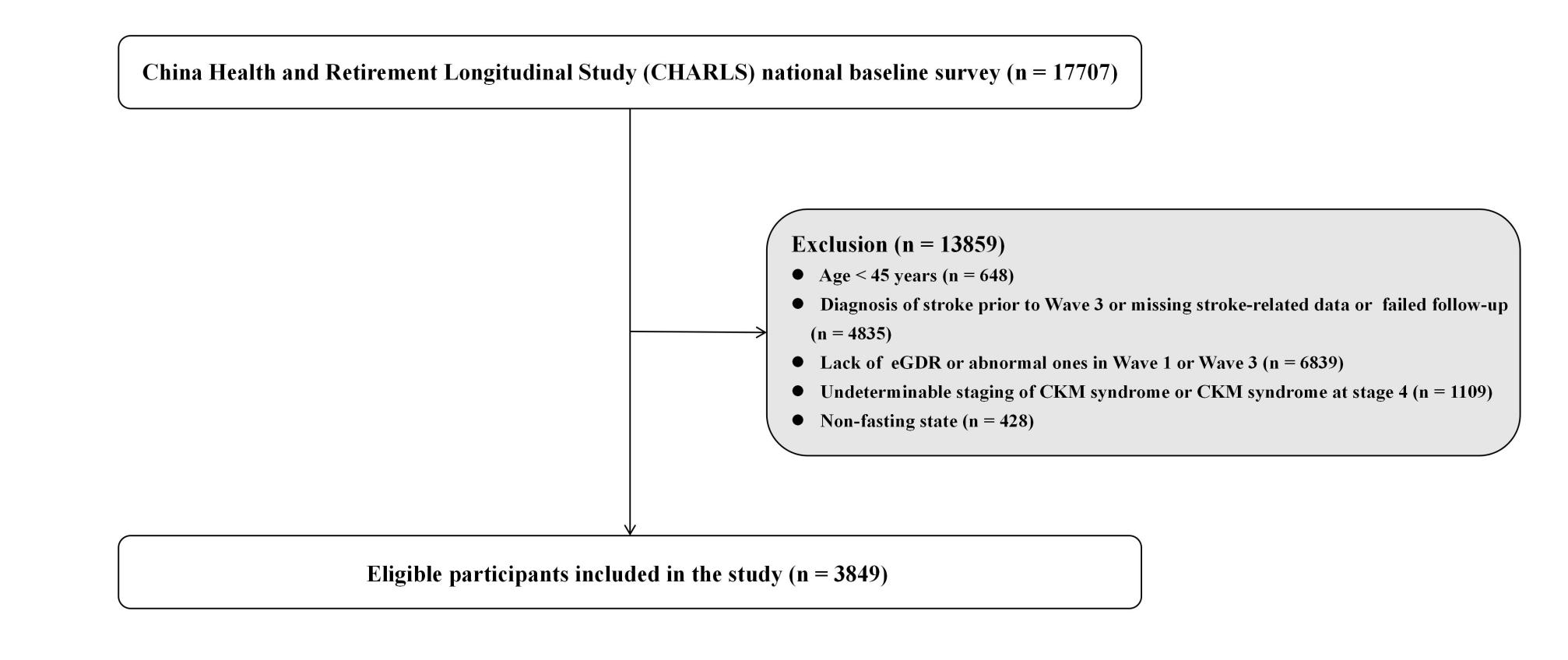
**

**Figure S1. Flow chart of study participants.**

**
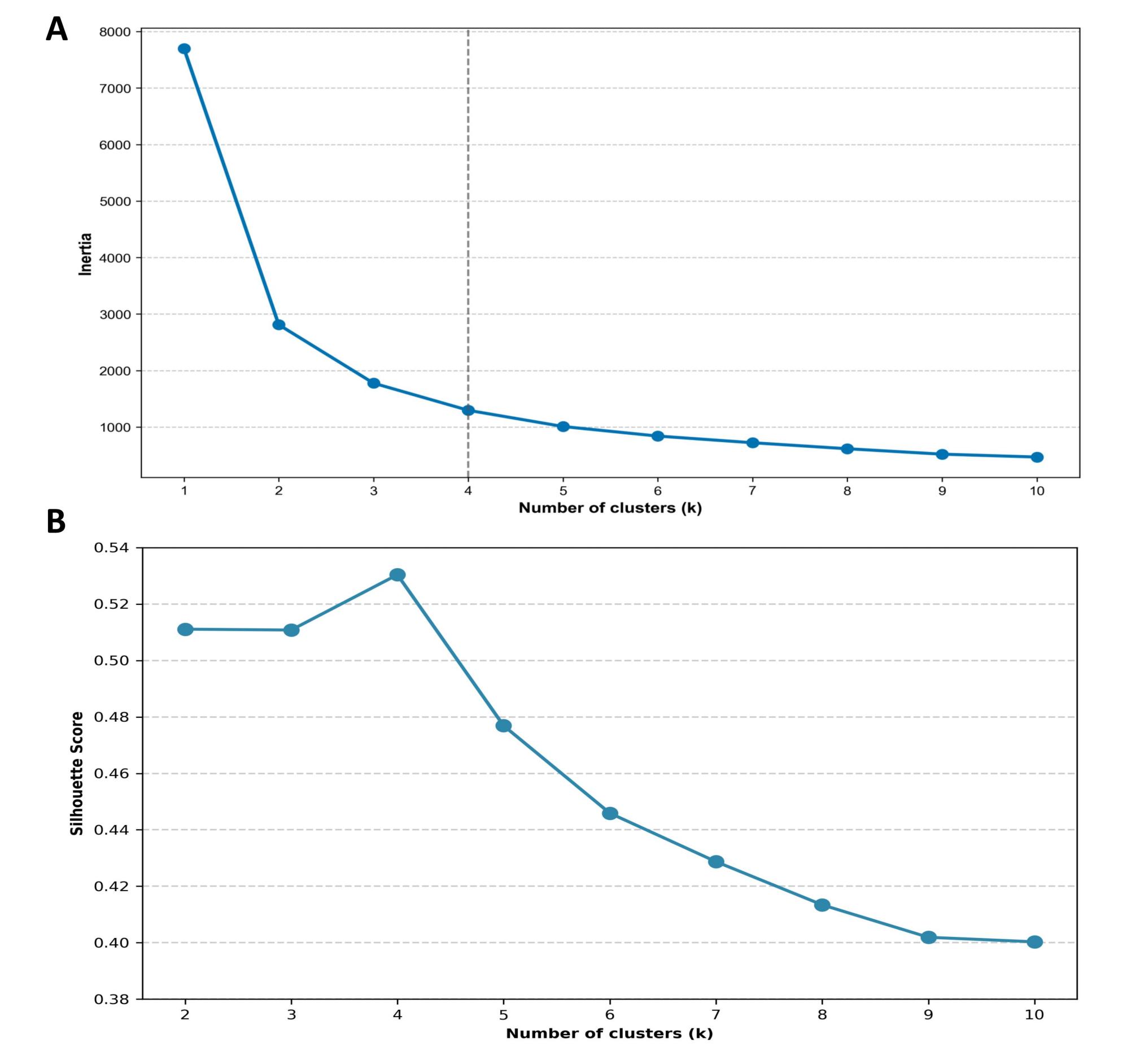
**

**Figure S2. Elbow method (A) and Silhouette Score (B) for determining the optimal number of clusters.**

**Supplementary Table S1**. Imputation of Missing Variables Based on Multiple Imputation Method

| Variable | Number |
| --- | --- |
| Drinking status | 4 |
| Smoking status | 9 |
| Dyslipidemia | 44 |
| LDL | 5 |
| Education | 1 |

**Supplementary Table S2**. Collinearity Analysis of Variables Based on Variance Inflation Factor

| Variable | VIF |
| --- | --- |
| Gender | 2.739 |
| Age | 1.772 |
| Education | 1.231 |
| Drinking status | 1.548 |
| Smoking status | 2.153 |
| Marital status | 1.086 |
| Habitation | 1.065 |
| BMI | 1.016 |
| TG | 1.508 |
| HDL | 1.313 |
| LDL | 1.086 |
| CRP | 1.017 |
| UA | 1.532 |
| eGFR | 1.594 |
| FBG | 1.152 |
| SBP | 2.371 |
| DBP | 2.236 |

**Supplementary Table S3.** Characteristics of the study participants based on the cumulative eGDR

SBP, systolic blood pressure; DBP, diastolic blood pressure; WC, waist circumference; BMI, body mass index; CRP, C-reactive protein; HDL-C, high-density lipoprotein cholesterol; LDL-C, low-density lipoprotein cholesterol; TC, total cholesterol; TG, triglyceride; BUN, blood urea nitrogen; UA, uric acid; FBG, fasting blood glucose; HbA1c, glycated hemoglobin; eGFR, estimated glomerular filtration rate; eGDR, estimated glucose disposal rate; CumeGDR, cumulative estimated glucose disposal rate;

| Characteristic | Cumulative eGDR | | | *P* value |
| --- | --- | --- | --- | --- |
|  | T 3 | T 2 | T 1 |  |
|  | (n=1283) | (n=1283) | (n=1283) |  |
| Age, years | 56.77±8.23 | 58.64±8.71 | 59.88±8.82 | <0.001 |
| Gender, n (%) |  |  |  | 0.059 |
| Male | 614 (47.9) | 631 (49.2) | 572 (44.6) |  |
| Female | 669 (52.1) | 652 (50.8) | 711 (55.4) |  |
| Marital status, n (%) |  |  |  | 0.066 |
| Married | 1176 (91.7) | 1161 (90.5) | 1141 (88.9) |  |
| Others | 107 (8.3) | 122 (9.5) | 142 (11.1) |  |
| Education, n (%) |  |  |  | 0.179 |
| Primary school or below | 874 (68.1) | 908 (70.8) | 914 (71.2) |  |
| Above primary school | 409 (31.9) | 375 (29.2) | 369 (28.8) |  |
| Habitation, n (%) |  |  |  | <0.001 |
| Agriculture | 916 (71.4) | 866 (67.5) | 816 (63.6) |  |
| Others | 367 (28.6) | 417 (32.5) | 467 (36.4) |  |
| Smoking status, n (%) |  |  |  | 0.001 |
| Never | 777 (60.6) | 764 (59.5) | 819 (63.8) |  |
| Previous | 82 (6.4) | 94 (7.3) | 113 (8.8) |  |
| Current | 424 (33) | 425 (33.1) | 351 (27.4) |  |
| Drinking status, n (%) |  |  |  | <0.001 |
| Never | 773 (60.2) | 718 (56) | 760 (59.2) |  |
| Previous | 80 (6.2) | 87 (6.8) | 117 (9.1) |  |
| Current | 430 (33.5) | 478 (37.3) | 406 (31.6) |  |
| Comorbidities, n (%) |  |  |  |  |
| Diabetes | 75 (5.8) | 166 (12.9) | 311 (24.2) | <0.001 |
| Dyslipidemia | 51 (4) | 86 (6.7) | 160 (12.5) | <0.001 |
| SBP, mmHg | 115.72±11.06 | 130.28±18.92 | 146.61±20.31 | <0.001 |
| DBP, mmHg | 68.88±8.65 | 75.85±11.27 | 84.18±12.11 | <0.001 |
| WC, cm | 79.01±6.70 | 83.88±9.42 | 91.93±7.97 | <0.001 |
| BMI, kg/m^2^ | 21.44±2.49 | 23.02±3.34 | 25.51±3.26 | <0.001 |
| CRP, mg/L | 0.76[1.15] | 0.91[1.25] | 1.26[1.85] | <0.001 |
| HDL, mg/dL | 54.47±15.04 | 52.14±15.73 | 47.68±13.90 | <0.001 |
| LDL, mg/dL | 113.71±31.89 | 116.17±33.98 | 121.61±37.74 | <0.001 |
| TC, mg/dL | 187.67±36.03 | 193.23±36.90 | 201.23±39.58 | <0.001 |
| TG, mg/dL | 87.61[54.87] | 100.00[74.78] | 124.79[95.14] | <0.001 |
| BUN, mg/dL | 15.72±4.35 | 15.64±4.48 | 15.64±4.23 | 0.871 |
| UA, mg/dL | 4.14±1.09 | 4.37±1.22 | 4.57±1.27 | <0.001 |
| FBG, mg/dL | 100.14±15.51 | 107.09±27.42 | 117.88±45.90 | <0.001 |
| HbA1c, % | 5.08±0.41 | 5.22±0.70 | 5.50±1.09 | <0.001 |
| eGFR, mL/min/1.73m² | 94.28±14.30 | 92.63±15.48 | 89.75±16.25 | <0.001 |
| CKM stage, n (%) |  |  |  | <0.001 |
| 0 | 214 (16.7) | 40 (3.1) | 1 (0.1) | <0.001 |
| 1 | 504 (39.3) | 210 (16.4) | 10 (0.8) |  |
| 2 | 388 (30.2) | 659 (51.4) | 745 (58.1) |  |
| 3 | 177 (13.8) | 374 (29.2) | 527 (41.1) |  |
| eGDR_2012_ | 11.25±0.64 | 9.55±1.30 | 6.62±1.02 | <0.001 |
| eGDR_2015_ | 10.76±0.74 | 7.79±1.11 | 5.91±1.02 | <0.001 |
| CumeGDR | 33.01±1.74 | 26.01±2.43 | 18.79±2.60 | <0.001 |
| Stroke, n (%) | 43 (3.4) | 100 (7.8) | 142 (11.1) | <0.001 |

**Table S4**. Incremental predictive value of cumeGDR and clusters of eGDR changes for stroke.

| Model | C-statistic (95%CI) | NRI (95% CI) | IDI (95% CI) |
| --- | --- | --- | --- |
| Basic model | 0.6574 (0.6352, 0.6993) | Reference | Reference |
| Basic model + cumeGDR | 0.6734 (0.6499, 0.7114) | 0.2342 (0.1033, 0.3457) | 0.0038 (0.0005, 0.0099) |
| Basic model + clusters of eGDR changes | 0.6711 (0.6480, 0.7105) | 0.2693 (0.1116, 0.3912) | 0.0032 (0.0009, 0.0095)  ) |

The basic model (model 4) included: age, sex, residence, educational level, marital status, alcohol consumption, smoking status, SBP, DBP, BMI, eGFR, TG, HDL-C, LDL-C, CRP, UA, and FBG.

**Table S5.** Subgroup analysis for the association between clusters of eGDR changes and stroke.

| Variable | The eGDR cluster groups, OR (95% CI) | | | |  |
| --- | --- | --- | --- | --- | --- |
|  | **Class 1** | **Class 2** | **Class 3** | **Class 4** | *P* for interaction |
| Age |  |  |  |  | 0.919 |
| <60 | Reference | 1.51 (0.86-2.67) | 2.31 (1.20-4.44) | 1.44 (0.81-2.55) |  |
| ≥60 | Reference | 1.79 (0.94-3.39) | 2.05 (1.03-4.06) | 2.66 (1.42-4.97) |  |
| Gender |  |  |  |  | 0.607 |
| Male | Reference | 1.30 (0.72-2.35) | 2.06 (1.09-3.89) | 1.75 (1.00-3.07) |  |
| Female | Reference | 1.68 (0.96-2.93) | 2.13 (1.16-3.91) | 1.83 (1.03-3.24) |  |
| Marital status |  |  |  |  | 0.772 |
| Married | Reference | 1.35 (0.87-2.09) | 1.96 (1.23-3.13) | 1.89 (1.25-2.87) |  |
| Others | Reference | 2.51 (0.82-7.68) | 3.72 (0.89-15.52) | 0.67 (0.13-3.52) |  |
| Education |  |  |  |  | 0.208 |
| Primary school or below | Reference | 1.83 (1.15-2.92) | 2.12 (1.26-3.57) | 2.04 (1.29-3.24) |  |
| Above primary school | Reference | 0.66 (0.27-1.57) | 1.55 (0.67-3.57) | 1.10 (0.48-2.51) |  |
| Habitation |  |  |  |  | 0.762 |
| Agriculture | Reference | 1.64 (1.02-2.63) | 1.92 (1.13-3.28) | 2.06 (1.31-3.24) |  |
| Others | Reference | 1.25 (0.56-2.80) | 2.41 (1.10-5.31) | 1.31 (0.56-3.08) |  |
| Smoking status |  |  |  |  | 0.989 |
| Never | Reference | 1.48 (0.88-2.49) | 1.64 (0.91-2.94) | 1.47 (0.86-2.52) |  |
| Smokers | Reference | 1.55 (0.81-2.96) | 2.32 (1.14-4.74) | 2.23 (1.21-4.11) |  |
| Drinking status |  |  |  |  | \| 0.945 \| \| --- \| |
| Never | Reference | 1.89 (1.09-3.26) | 2.68 (1.50-4.81) | 2.08 (1.21-3.58) |  |
| Alcohol drinkers | Reference | 1.12 (0.61-2.05) | 1.52 (0.78-2.96) | 1.52 (0.84-2.75) |  |
| CKM stage |  |  |  |  | 0.599 |
| 0-1 | Reference | 1.42 (0.17-11.79) | 2.91(0.30-28.45) | 1.73 (0.79-3.76) |  |
| 2 | Reference | 1.65 (1.15-2.38) | 2.38 (1.62-3.53) | 1.46 (0.98-2.18) |  |
| 3 | Reference | 1.63 (0.70-3.76) | 2.62 (1.04-6.57) | 2.97 (1.26-7.01) |  |

**The analysis was adjusted using Model 4.**

**Table S6.** Subgroup analysis for the association between cumulative eGDR and stroke

| Variable | cumulative eGDR, OR (95% CI) | | |  |
| --- | --- | --- | --- | --- |
|  | **T3** | **T2** | **T1** | *P* for interaction |
| Age |  |  |  | 0.829 |
| <60 | Reference | 2.03 (1.19-3.44) | 2.65 (1.40-5.03) |  |
| ≥60 | Reference | 2.72 (1.43-5.16) | 3.11 (1.57-6.18) |  |
| Gender |  |  |  | 0.818 |
| Male | Reference | 2.24 (1.30-3.86) | 2.38 (1.28-4.44) |  |
| Female | Reference | 2.14 (1.23-3.71) | 3.17 (1.74-5.80) |  |
| Marital status |  |  |  | 0.171 |
| Married | Reference | 2.27 (1.50-3.44) | 2.69 (1.69-4.29) |  |
| Others | Reference | 1.57 (0.52-4.74) | 4.15 (1.14-15.15) |  |
| Education |  |  |  | 0.993 |
| Primary school or below | Reference | 2.27 (1.45-3.54) | 2.85 (1.73-4.70) |  |
| Above primary school | Reference | 1.73 (0.79-3.81) | 2.12 (0.87-5.13) |  |
| Habitation |  |  |  | 0.678 |
| Agriculture | Reference | 2.36 (1.53-3.66) | 2.59 (1.57-4.29) |  |
| Others | Reference | 1.95 (0.85-4.45) | 3.48 (1.46-8.34) |  |
| Smoking status |  |  |  | 0.675 |
| Never | Reference | 1.96 (1.18-3.26) | 2.49 (1.39-4.46) |  |
| Smokers | Reference | 2.47 (1.36-4.50) | 2.74 (1.38-5.46) |  |
| Drinking status |  |  |  | 0.362 |
| Never | Reference | 2.91 (1.70-4.97) | 4.10 (2.26-7.43) |  |
| Alcohol drinkers | Reference | 1.52 (0.87-2.66) | 1.72 (0.91-3.24) |  |
| CKM stage |  |  |  | 0.764 |
| 0-1 | Reference | 2.76 (1.33-5.73) | 2.82 (0.30-26.47) |  |
| 2 | Reference | 1.43 (0.80-2.56) | 2.14 (1.16-3.92) |  |
| 3 | Reference | 3.66 (1.37-9.75) | 3.70 (1.33-10.29) |  |

**The analysis was adjusted using Model 4.**

**Supplementary Table S7.** Sensitivity analysis of the association between different eGDR groups and stroke after excluding the interpolation participants

| Variable | Model 1 | | Model 2 | | Model 3 | | Model 4 | |
| --- | --- | --- | --- | --- | --- | --- | --- | --- |
|  | OR (95% CI) | *P* value | OR (95% CI) | *P* value | OR (95% CI) | *P* value | OR (95% CI) | *P* value |
| Categories |  |  |  |  |  |  |  |  |
| Class 1 | Reference |  | Reference |  | Reference |  | Reference |  |
| Class 2 | 1.91 (1.36-2.69) | <0.001 | 1.76 (1.25-2.49) | 0.001 | 1.74 (1.23-2.46) | 0.001 | 1.51 (1.01-2.25) | 0.048 |
| Class 3 | 3.03 (2.17-4.22) | <0.001 | 2.84 (2.03-3.97) | <0.001 | 2.91 (2.08-4.08) | <0.001 | 2.03 (1.31-3.15) | <0.001 |
| Class 4 | 1.92 (1.30-2.85) | 0.001 | 1.87 (1.26-2.77) | 0.002 | 1.87 (1.26-2.77) | 0.002 | 1.76 (1.18-2.62) | 0.006 |
| **Tertiles** |  |  |  |  |  |  |  |  |
| T 3 | Reference |  | Reference |  | Reference |  | Reference |  |
| T 2 | 2.43 (1.68-3.50) | <0.001 | 2.34 (1.62-3.37) | <0.001 | 2.34 (1.62-3.39) | <0.001 | 2.16 (1.47-3.18) | <0.001 |
| T 1 | 3.49 (2.46-4.96) | <0.001 | 3.29 (2.31-4.69) | <0.001 | 3.36 (2.35-4.80) | <0.001 | 2.66 (1.72-4.10) | <0.001 |
| *P* for trend |  | <0.001 |  | <0.001 |  | <0.001 |  | <0.001 |
| **CumeGDR**^*^ | 0.64 (0.56-0.72) | <0.001 | 0.65 (0.57-0.74) | <0.001 | 0.64 (0.57-0.73) | <0.001 | 0.71 (0.60-0.85) | <0.001 |

^*^ Per SD.

**Supplementary Table S8.** Sensitivity analysis of the association between different eGDR groups and stroke after excluding participants who died within 3 years

| Variable | Model 1 | | Model 2 | | Model 3 | | Model 4 | |
| --- | --- | --- | --- | --- | --- | --- | --- | --- |
|  | OR (95% CI) | *P* value | OR (95% CI) | *P* value | OR (95% CI) | *P* value | OR (95% CI) | *P* value |
| Categories |  |  |  |  |  |  |  |  |
| Class 1 | Reference |  | Reference |  | Reference |  | Reference |  |
| Class 2 | 1.62 (1.25-2.09) | <0.001 | 1.57 (1.20-2.04) | <0.001 | 1.59 (1.22-2.07) | <0.001 | 1.68 (1.28-2.23) | <0.001 |
| Class 3 | 2.38 (1.83-3.09) | <0.001 | 2.27 (1.74-2.96) | <0.001 | 2.31 (1.77-3.02) | <0.001 | 2.65 (1.86-3.76) | <0.001 |
| Class 4 | 1.70 (1.26-2.30) | <0.001 | 1.72 (1.27-2.32) | <0.001 | 1.72 (1.27-2.33) | <0.001 | 1.77 (1.30-2.42) | <0.001 |
| **Tertiles** |  |  |  |  |  |  |  |  |
| T 3 | Reference |  | Reference |  | Reference |  | Reference |  |
| T 2 | 1.53 (1.18-1.98) | 0.001 | 1.51 (1.17-1.96) | 0.002 | 1.53 (1.18-1.98) | 0.001 | 1.60 (1.21-2.10) | <0.001 |
| T 1 | 2.15 (1.68-2.76) | <0.001 | 2.06 (1.60-2.64) | <0.001 | 2.08 (1.62-2.68) | <0.001 | 2.30 (1.66-3.17) | <0.001 |
| *P* for trend |  | <0.001 |  | <0.001 |  | <0.001 |  | <0.001 |
| **CumeGDR**^*^ | 0.72 (0.56-0.93) | 0.012 | 0.74 (0.57-0.95) | 0.019 | 0.73 (0.56-0.94) | 0.016 | 0.71 (0.53-0.94) | 0.016 |

^*^ Per SD.
